# Translational perspectives on women’s mental health

**DOI:** 10.1192/bjp.2025.35

**Published:** 2025-06

**Authors:** Liisa A. M. Galea

**Affiliations:** Treliving Family Chair in Women’s Mental Health, Centre for Addiction and Mental Health, Department of Psychiatry, University of Toronto, Ontario, Canada

**Keywords:** Animal models, menopause, menopausal hormone therapy, perinatal psychiatry, sex differences

## Abstract

Preclinical and clinical research have devoted limited attention to women’s health. Animal models centred on female-specific factors will improve our understanding of mental health disorders. Exploring the heterogeneity of mental health disorders, in concert with attention to female-specific factors, will accelerate the discovery of efficacious treatments for mental health disorders.

Randomised clinical trials have been hailed as the gold standard for testing medical interventions, yet 90% fail. With the high costs of bringing each new therapy to trial, the urgency in finding efficient paths to deliver effective treatments to trial has never been greater. It may also be time to abandon our ‘one size fits all’ approach to new therapeutics, particularly with respect to primary outcomes. Much has been written about the lack of preclinical studies in females or the lack of use of sex-based analyses to understand whether therapeutics have differential effects in improving symptoms by sex. However, less is written about within-sex influences on treatment efficacy. How do female-specific factors such as menstrual cycle phase or menopause status influence drug efficacy and/or disease risk? If only 3% of neuroscience studies are conducted using females,^1^ can we use animal modelling to fast-track our understanding of how women’s health factors can influence health outcomes and disease risk?

As a researcher who primarily uses preclinical models to explore our understanding of human disease, I know all too well the scepticism that comes with using animal models, particularly as it concerns mental health. Although depression, psychosis and anxiety are uniquely human, it is possible to model endophenotypes of disease in animal models. Using the example of major depressive disorder (MDD), certain symptoms of MDD can be modelled in rodents, including anhedonia, changes in appetite, sleep dysregulation, fatigue and changes in cognition. Although we cannot model depressed mood or thoughts of suicide, we can model changes in coping behaviours and probe neural biomarkers that are implicated in the pathophysiology of MDD.

The failure of translation from preclinical to clinical trials is probably due both to the failure of effective animal models and a failure of our understanding of the human disease. There is great variety in the symptom presentation of MDD, with >200 unique symptom combinations possible.^2^ There is also little overlap in the 52 symptoms assessed across 7 common scales used in clinical research,^3^ and thus it is important to consider whether the failure of good translation is due to the animal model itself or our lack of understanding of the particular human disease. Herein within-sex studies are crucial, because the consideration of the heterogeneity of disease is important in order to properly achieve precision medicine. Diagnostic manuals for mental disorders acknowledge that there are several subtypes of MDD, but primary and clinical research are not often directed to these subtypes.

Furthermore, because our diagnosis criteria are often based on the experiences of males, symptom scales do not always ask about the symptoms that are more typically seen in human females. For example, hyperphagia, somatic complaints and hypersomnia are more likely to be seen in women compared with men, yet questions on these symptoms are not present in most scales. Thus, our lack of attention to, and analyses of, sex differences in symptoms with diagnosis is probably affecting our progress. There is a call to improve rigour and reproducibility in the animal literature of human disease. Part of the impetus of sex as a biological variable is the idea that accounting for sex and related factors will improve rigour and reproducibility. Indeed, in many brain disorders, sex differences are noted in manifestation suggesting that different neural and molecular pathways are activated with certain disorders. Indeed, transcriptional analyses show either little overlap in genes expressed with MDD across multiple brain regions, differing numbers of transcripts by brain regions, differing cell types or gene expression patterns that change in opposing directions by sex.^4^ These differences in MDD gene expression patterns suggest mechanistic differences between the sexes, highlighting the need for personalised approaches in treatment. However, a sole focus on possible sex differences misses the point that within-sex comparisons are important. A fulsome understanding of women’s mental health will not be achieved until we understand how female-specific factors can also drive health outcomes and disease risk from animal models to clinical studies.

When building a model of disease, it is important to ask who is getting the disorder and when, because this will allow us to understand the disorder. Although more women than men are diagnosed with MDD, the time of greatest risk for initial diagnosis is in the postpartum. Perinatal depression (PND) is described as depression during pregnancy or in the 1 month following pregnancy. However, for building models of first-time depression, it is important to consider that the risk is seen during the first 3 months postpartum. Work by Alkistis Skalkidou demonstrates that there are a variety of patterns with depression onset during the early or late postpartum, or during pregnancy itself for PND.^5^ We created two animal models of postpartum depression (PPD) – one based on earlier onset and the other on later onset; each of these models has distinct effects on endophenotypes of depression and treatment efficacy.^6^ Other models target stress during pregnancy, modelling the depression seen during pregnancy, which has different effects on treatment efficacy. Each of these findings across models mirrors what is seen in the clinical literature, underscoring why it is so important to model the heterogeneity of PND.^6^ Several models are needed, because diversity in disease endophenotypes is seen also in human disease. Preclinical work by Jamie Macguire, who examined the link between allopregnanolone and depressive-like behaviours in the postpartum, found that by manipulating the GABA_A_ receptor, she was able to prevent the depressive-like endophenotypes in her model. These findings contributed to the preclinical evidence that led to successful clinical trials using synthetic allopregnanolone to treat PPD and, for the first time, US Food and Drug Authority approval for a drug specifically for PPD.

However, as noted above, one size does not fit all even for women’s mental health. Although allopregnanolone works for certain individuals with PPD, it does not work for all those with PPD, probably due to the heterogeneity of PPD as described above. It also does not appear to be an effective treatment for premenstrual dysphoric disorder.^7^ Although it is known that menopause, pregnancy, hormonal contraceptives and menopausal hormone therapy (MHT) can influence female health, it is less acknowledged that there are many menopauses, many pregnancies, many forms of PND and many formulations of MHT and hormonal contraceptives. All of these factors need to be studied in appropriate animal and clinical models.

How can our understanding of the overgeneralisation of women’s health help us understand the utility of animal models? An example can be seen in the literature surrounding the cognitive effects of MHT. Most animal studies show that MHT is beneficial for cognition and brain health, yet the findings in humans are equivocal.^8^ Is this just an indication that what happens in rodents is not recapitulated in humans? Upon closer observation, most animal models use a surgical menopause (ovariectomy) and subcutaneous administration of oestrogens, which is usually given shortly following ovariectomy. Taken together, this aligns well with findings in humans because: (a) surgical menopause is associated with greater risk of dementia; (b) MHT use is associated with improvements in cognition and reduced loss of hippocampal grey matter with surgical menopause; (c) the critical window hypothesis suggests that MHT is more beneficial when initiated closer to menopause; and (d) transdermal MHT is more beneficial than oral formulations for brain health. Thus, animal models can show good coherence with human findings when we take into account the details of the human condition and animal modelling. Although it has been argued that rodents do not undergo a typical human menopause, rats and mice do undergo reproductive senescence with reductions in fertility, increases in follicular stimulating and luteinising hormones and irregular cycling followed by cessation of cycling. Menopause subtypes can also be modelled in rodents with ovariectomy for surgical menopause or administration of pharmacological agents such as vinyl cyclohexine dioxide as models of induced (chemical) menopause. When translating findings, researchers need to consider menopause type, age, hormone formulation and route of administration. Thus, care must be taken to understand what is being modelled and how, because details matter in regard to understanding menopause effects on brain and cognition, with menopause type, MHT type and brain regions targeted all influencing findings.

Animal research offers benefits including control of diet, living condition and activity levels within a shorter lifespan, which reduces confounds that influence health. Importantly, there are advanced genetic and imaging tools available in animal models such that it is possible to examine the influence of particular genetic polymorphism, humanised genes or the specific contribution of particular neurons to a particular neural circuit in animal models. Using these tools can recapitulate what is seen in human conditions, particularly when sex and women’s health factors are taken into account.^9^ New research from our own laboratory indicates that a longer time following surgical menopause (ovariectomy) leads to reductions in putative neural stem cells. Is this one possible mechanism for why MHTs lose their effectiveness the longer the time spent following surgical menopause? Animal models can also help in understanding whether those neural stem cells are permanently lost over time or whether they are merely quiescent and, if so, what treatments may be used to rejuvenate the neural stem cell population to reverse or repair neurodegeneration with ageing.

To advance personalised medicine, we must embrace not only the heterogeneity of disease but also heterogeneity within our clinical and animal and models. Our animal models need to reflect the diversity of human disease, and diversity in our animal models is critical to understanding that diversity in disease. When animal models do not recapitulate our understanding of human health, we need to critically examine both the animal model along with our knowledge of human disease. Human mental health disorders are complex, and animal models can help unravel that complexity providing we adopt both heterogeneity within those models and pay attention to female health factors. Funding agencies need to prioritise funding calls for biomedical research in women’s health supporting animal models, clinical research and human trials. Good translation is possible with accelerated clinical success, but we must embrace the complexity of human disease and animal modelling. One size fits all impedes the progress of personalised medicine. Lecanamab, a therapeutic recently approved for Alzheimer’s disease, favours cognitive improvement in human males with this condition, but not in human females. We will be closer to finding effective treatments for women’s mental health when we prioritise it using appropriate models. Research using female modelling is scarce: only 3% of studies were conducted solely in females, and under 2% in neuroscience have centred on hormonal contraceptives, menopause, MHT, pregnancy and menstrual cycles. We all need to do better. All of us have a role to play in moving medical research forward: funders, publishers, reviewers and researchers need to prioritise women’s mental health research to achieve the promise of personalised medicine and, importantly, health equity.

## Data Availability

Data availability is not applicable to this article as no new data were created or analysed in this study.
